# Altering the linker in processive GH5 endoglucanase 1 modulates lignin binding and catalytic properties

**DOI:** 10.1186/s13068-018-1333-3

**Published:** 2018-12-18

**Authors:** Zhen Wang, Tianrui Zhang, Liangkun Long, Shaojun Ding

**Affiliations:** The Co-Innovation Center of Efficient Processing and Utilization of Forest Resources, Jiangsu Key Lab for the Chemistry & Utilization of Agricultural and Forest Biomass, College of Chemical Engineering, Nanjing Forestry University, Nanjing, 210037 Jiangsu China

**Keywords:** GH5 endoglucanase, Processive enzyme, Lignin binding, Polypeptide linker, Quartz crystal microgravimetry

## Abstract

**Background:**

The non-productive adsorption of cellulases onto lignin in biomass is a key issue for the biofuel process economy. It would be helpful to reduce the inhibitory effect of lignin on enzymatic hydrolysis by engineering weak lignin-binding cellulases. Cellulase linkers are highly divergent in their lengths, compositions, and glycosylations. Numerous studies have revealed that linkers can facilitate optimal interactions between structured domains. Recently, efforts have focused on the contributions and mechanisms of carbohydrate-binding modules and catalytic domains that affect lignin affinity and processivity of cellulases, but our understanding of the effects of the linker regions on lignin adsorption and processivity of GH5 processive endoglucanases is still limited.

**Results:**

Eight GH5 endoglucanase 1 variants of varying length, flexibility, and sequence in the linker region were constructed. Their characteristics were then compared to the wild-type enzyme (EG1). Remarkably, significant differences in the lignin adsorption profiles and processivities were observed for EG1 and other variants. Our studies suggest that either the length or the specific amino acid composition of the linker has a prominent influence on the lignin-binding affinity of the enzymes. Comparatively, the processivity may depend primarily on the length of the linker and less so on the specific amino acid composition. EG1-ApCel5A, a variant with better performance in enzymatic hydrolysis in the presence of lignin, was obtained by replacing a longer, flexible linker. In total, up to between 28.2 and 30.1% more reducing sugars were generated from filter paper by EG1-ApCel5A in the presence of lignin compared to EG1.

**Conclusions:**

Our results highlight the relevance of the linker region in the lignin adsorption and processivity of a processive endoglucanase. Our findings suggest that the linker region may be used as a target for the design of more active and weaker lignin-binding cellulases.

**Electronic supplementary material:**

The online version of this article (10.1186/s13068-018-1333-3) contains supplementary material, which is available to authorized users.

## Background

The conversion of lignocellulosic biomass into biofuels is a promising way to reduce the demand for non-renewable fossil fuels [1, 2]. Enzymatic saccharification of cellulose in lignocellulose into glucose is a crucial step in the economical production of biofuels via biological-mediated conversion processes. Complete hydrolysis of cellulose to glucose requires the synergistic action of three kinds of glycosyl hydrolases, endoglucanases (EGs; EC 3.2.1.4), cellobiohydrolases (CBHs; EC 3.2.1.91), and β-glucosidases (EC 3.2.1.21) [1]. In addition, oxidative enzymes, namely lytic polysaccharide monoxygenases (LPMOs), are required [3–6]. However, low enzymatic hydrolysis efficiencies and the necessity of high dosages of cellulase for the hydrolysis of pretreated lignocellulosic biomasses are major impediments to the commercialization of lignocellulosic biofuels [7, 8].

The non-productive adsorption of cellulases onto lignin in biomass is a key economic issue for biofuel processing, since higher cellulase loadings are required to overcome the inhibitory effect of lignin, and cellulase recycling is made difficult after the hydrolysis reaction [9–13]. Many cellulases are multi-domain enzymes consisting of a catalytic domain (CD) and a carbohydrate-binding module (CBM) connected by a flexible linker peptide. The primary role of CBMs in cellulases is to recognize and specifically bind to insoluble cellulose, thereby increasing the effective enzyme concentration on the cellulose surface and enhancing enzymatic activity [14]. CBMs are also believed to be responsible for the majority of lignin binding through hydrophobic interactions [15–18]. The non-productive binding of enzymes to lignin is also influenced by features specific to the CD. Accordingly, the CDs of different GH5 family members display different affinities for lignin, with binding being driven by electrostatic interactions or hydrogen bonding depending on the enzyme in question [10, 12, 19]. Many efforts have been devoted to the prevention of non-productive adsorption in enzymatic hydrolysis, such as changing pretreatment conditions [20, 21] or blocking the exposed lignin surface using bovine serum albumin (BSA) and additives [22–24]. A promising alternative approach would be engineering or discovering natural cellulases with low lignin-binding propensities, relying on a full understanding of enzyme–lignin interactions [17].

The term “processive” can be attributed to enzymes that remain attached to their substrates and perform multiple rounds of catalysis before dissociating [25]. Current research shows that there are two kinds of processive cellulases, including cellobiohydrolases and processive endoglucanase. Unlike cellobiohydrolases, classic endoglucanases randomly hydrolyze the β-1,4-glycosidic bonds in the interior of the chains. However, some bacteria and fungi produce so-called processive endoglucanases. These bind to the glucan chains, introduce internal bond breakages, and release soluble cellooligosaccharides before desorption [26–28]. In this regard, CBMs have been shown to crucially contribute to cellulase processivity. The deletion of the CBM3a module in the processive Cel9A-90 endoglucanase from *Thermomonospora fusca* leads to the loss of processivity and the inability to degrade crystalline cellulose [29]. In our previous work, the removal of the CBM1 module significantly reduced the processivity of a GH5 processive endoglucanase EG1 from *Volvariella volvacea* [27]. However, the addition of an extra CBM1 to the non-modular GH5 endoglucanase significantly improved not only its binding affinity and catalytic activity to insoluble celluloses but also its processivity [30].

Although lignin adsorption and processivity are two distinct properties of cellulases, both appear to be highly related to the properties of the CBM and its synergistic interplay with the catalytic domain. Compared to the high homology of family-1 CBMs in fungal cellulases, linkers are highly divergent in their lengths, compositions and glycosylations [31]. Numerous studies have revealed that linkers can facilitate optimal interactions between structured domains [31]. Recently, efforts have focused on the contribution and mechanism of CBMs to the lignin affinity and processivity of cellulases, yet knowledge about the involvement of the linker region in lignin adsorption and processivity is still scarce. Thus, a processive GH5 endoglucanase, EG1 (GenBank No. AF329732) from *V. volvacea*, was selected in this study [27]. Eight EG1 variants of different length, flexibility, and sequence in the linker region were constructed, and their characteristics were compared to the wild-type enzyme for comprehensive analyses of their effects on lignin adsorption and catalysis. The hydrolytic efficiencies of variants with variations in their linker regions were also tested in the presence of lignin to investigate the correlation between lignin affinity and lignin inhibition. These results indicate that the linker region may be used as a target for the design of cellulases with higher catalytic activity and weaker lignin binding.

## Methods

### Bacterial and yeast hosts, culture media, and chemicals

*Escherichia coli* Top10 and *Pichia pastoris* strain KM71H were used for plasmid construction and propagation, and the expression of EG1, respectively. Yeast culture media were prepared according to the manual for the *Pichia* expression system from Invitrogen (San Diego, CA). All chemicals were of analytical grade or higher and purchased from Sigma (St. Louis, MO) unless otherwise indicated.

### Construction of plasmids

The plasmid pPICZαB-EG1 containing an entire *eg1* gene was used as the template for variant construction. The DNA fragment encoding CBM-deleted variant EG1CD was amplified by PCR with the primers, as shown in Table 1. The amplified DNA fragment was ligated into the *Pst*I and *Xba*I sites of the pPICZαB vector (Invitrogen) to yield the expression vector pPICZαB-CD. The DNA fragments encoding EG1-Δ10 and EG1-Δ19 variants, with 10-amino acid (TTTSSAPNPT) and 29-amino acid (GPTTTSSAPNPTSSGCPNA) deletions in the linker region of EG1, respectively, were constructed using overlap PCR in two steps. First, the fragments encoding the CBM with a partial region of the linker and the CD with a partial region of the linker were amplified individually by PCR using pPICZαB-EG1 as a template with the primers listed in Additional file 1: Table S1; second, the two fragments were fused by overlap PCR. *Pst*I and *Xba*I restriction enzyme sites were introduced to the 5′ and 3′ ends of the EG1-Δ10 and EG1-Δ19 fragments, respectively. After digestion, the fragments were ligated at the *Pst*I/*Xba*I sites of the pPICZαB *Pichia* expression vector to yield the expression plasmids pPICZαB-EG1-Δ10 and pPICZαB-EG1-Δ19. The DNA fragments encoding variants EG1-A(EAAAK)_2_A, EG1-ApCel5A, and EG1-L1 were also amplified by overlap PCR by the same protocol using pPICZαB-EG1 as a template and the primers, listed in Table 1. After digestion with *Pst*I and *Xba*I restriction enzymes, the fragments were ligated at the *Pst*I/*Xba*I sites of the pPICZαB *Pichia* expression vector to yield the expression plasmids pPICZαB-EG1-A(EAAAK)_2_A, pPICZαB-EG1-ApCel5A, and pPICZαB-EG1-L1, respectively. Site-directed mutations in EG1(P→G) and EG1(G→P) were carried out by PCR using primers with selected site mutations (Additional file 1: Table S1) and the pPICZαB-EG1 plasmid as the template. The PCR products were purified from gels and treated with *Dpn*I to eliminate the template plasmids. All EG1 and engineered variants contained a 6-histidine tag at the C-terminus to facilitate purification using metal-affinity chromatography.

**Table 1:** Specific activities of EG1and its variants on different substrates

Enzymes	Specific activity (U/μmol)		
	CMC-Na	RAC	FP
EG1	1480.57 ± 30.99	574.20 ± 19.09	8.10 ± 0.12
EG1-Δ10	1164.83 ± 46.52	454.18 ± 20.51	5.66 ± 0.22
EG1-Δ19	235.10 ± 9.06	150.62 ± 6.05	4.35 ± 0.14
EG1-A(EAAAK)_2_A	1501.36 ± 45.69	422.48 ± 11.04	7.36 ± 0.15
EG1CD	1350.09 ± 49.62	477.17 ± 21.09	7.00 ± 0.11
EG1-ApCel5A	1646.48 ± 46.92	572.17 ± 5.29	7.51 ± 0.28
EG1-L1	1464.64 ± 23.50	476.94 ± 11.96	7.29 ± 0.16
EG1-(P→G)	1288.30 ± 11.15	342.98 ± 12.33	6.60 ± 0.39
EG1-(G→P)	1594.39 ± 49.83	550.00 ± 27.55	8.95 ± 0.16

Values shown are means of triplicate determinations ± standard error (SE)

### Expression and purification of EG1 and its variants

The plasmids were linearized using *Sac*I and transformed into *P. pastoris* KM71H competent cells by electroporation according to the manual of the *Pichia* expression system from Invitrogen. *P. pastoris* transformants were induced and harvested as described previously [27]. Supernatants from 25-mL cultures were collected by centrifugation (6500*g* for 10 min). The crude enzymes were purified by affinity chromatography using Ni–NTA Agarose gel (Qiagen, Valencia, CA, USA) according to the manufacturer’s manual. The molecular weight and enzyme purity of the purified protein were estimated by sodium dodecyl sulfate-polyacrylamide gel electrophoresis (SDS-PAGE) (10% wt/vol.).

### Biochemical and processive properties of EG1 and its variants

Enzyme activities against soluble or insoluble celluloses were assayed by measuring the amount of reducing sugars released from various substrates. The substrates tested were CMC-Na, regenerated amorphous cellulose (RAC), and filter paper (FP, Whatman, Little Chalfont, UK). RAC was prepared from Avicel PH-101 as previously described [32]. The assay mixtures contained 0.9 mL of potassium phosphate buffer (100 mM, pH 7.5), a 0.5 mL suspension of FP (50 mg) or a 0.5 mL solution of substrate (2% for CMC-Na, 1% for RAC), and 0.1 mL of appropriately diluted enzyme sample (2 μg for CMC-Na and RAC, 100 μg for FP). The reaction was carried out at 50 °C for 30 min (60 min for FP). The released reducing sugars were measured at 520 nm by the Somogyi–Nelson method. Each assay was performed in triplicate. One unit of enzymatic activity was defined as the amount of enzyme that released 1 μmol of reducing sugar equivalent per min under the assay conditions described.

Processivity was assayed by measuring the ratio of soluble to insoluble reducing sugars derived from FP and RAC as described previously [33]. Enzymatic reactions were performed under the assay conditions. The supernatants and residues were separated by centrifugation. The amounts of reducing sugars in the supernatants and in the residues of FP strips and RAC (after the latter had been washed three times with reaction buffer and resuspended in the initial volume of buffer) were measured by the Somogyi–Nelson method.

### Lignin preparation

Dry Masson pine was milled in a planetary ball mill (Fritsch Pulverisette 7, Idar-Oberstein, Germany) at 40 °C as described previously [34]. The milled Masson pine was dispersed in a dioxane/water (96/4, v/v) mixture in a 1:25 ratio of wood to solvent and mechanically stirred. After 1 day, the suspension was centrifuged and the residue redispersed in a fresh dioxane:water mixture and stirred for an additional day. The extracts after two rounds of extraction were combined and then freeze dried to give crude milled Masson pine lignin (MMPL) with roughly a 20–30% yield; this lignin contains up to about 10% residual carbohydrate. The crude MMPL was further purified by dissolving the lignin in 90% acetic acid (20 mL for each gram of MMPL). The acetic acid solution was then added dropwise to water while stirring. The precipitated lignin was centrifuged and then air dried in a rotary vacuum evaporator. It was then dissolved with stirring in a mixture of 1,2-dichloro-ethane:ethanol (2:1, v:v) and centrifuged to remove the insoluble residues. The lignin solution was added dropwise to anhydrous ethyl ether to precipitate the lignin. After centrifugation, the insoluble purified MMPL was washed three times with fresh ethyl ether. The yield of the purified MMPL was approximately half that of the crude preparation, but its residual carbohydrate content was about 4%. The Masson pine kraft lignin (MPKL) was isolated from kraft pulping black liquid by precipitating the lignin at an acidic condition (pH 4) adjusted using 20–30% (w/v) of sulfuric acid. The precipitated lignin was then separated by filtration, extensively washed with distilled water to a neutral pH, and air dried in a vacuum drying oven.

### Lignin-binding analysis

The enzyme adsorption/desorption behaviors on lignin films were monitored in situ using quartz crystal microgravimetry with dissipation monitoring (QCM-D) (QCM-D E4 model, Biolin Corp., Gothenburg, Sweden) at 4 °C as previously described [34]. The purified MMPL was dissolved in dioxane at a concentration of 1% (w/v). The solution was coated on QCM gold sensors with a spin coater (KW-4A, Shanghai Daojing Instrument Co., Ltd., Shanghai, China) operated at 3000 rpm for 1 min. The coating was repeated three times to ensure full coverage of the sensor surface. Before the measurement, potassium phosphate buffer (100 mM, pH 7.5) was filled into the measuring chambers by a peristaltic pump at a flow rate of 0.1 mL/min until the frequency and temperature stabilized. Thereafter, cellulase solution (protein concentration 0.05 mg/mL) was injected at the same flow rate of 0.1 mL/min until reaching stable conditions. The frequency change was then tracked. Buffer solution was introduced again to rinse the system after a plateaued signal. The temperature was maintained at 4 °C in all experiments, with each condition tested at least in triplicate. All measurements were recorded at a 5 MHz fundamental resonance frequency with its overtones corresponding to 15, 25, 35, and 45 MHz. The third overtone (15 MHz) was used for data processing.

QCM functions as a microbalance because the negative change in the resonance frequency, Δ*f* (Δƒ = *f* − *f*_o_), of an oscillating QCM sensor is proportional to the change in mass, Δ*m*, of the QCM sensor as derived by Sauerbrey [35]. The Sauerbrey equation (Eq. 1) was adopted in this work to estimate the adsorbed mass of enzymes on lignin film.

1 $$\Delta m = - C\frac{\Delta f}{n},$$

where *n* is the overtone order and *C* is the mass sensitivity constant. For crystals with *f*_o_ = 5 MHz, *C* is 17.7/Hz ng/cm^2^.

### Influence of lignin on activity and hydrolysis of EG1 and its variants

Two types of lignin (MMPL and MPKL) were used, respectively, to evaluate their inhibitory effects on activity and hydrolysis of EG1 and its variants. Relative inhibition was defined as the percentage of decreased activities or hydrolysis efficiencies to corresponding values without lignin. The influence of lignin on the activities of EG1 and its variants was assayed under the same conditions used for the activity assay, except that lignin (2 mg/mL) was added to the reaction mixtures.

The inhibitory effect of lignin on enzymatic hydrolysis was studied by observing the hydrolysis of cellulose in the presence of purified MMPL or MPKL. Reactions were conducted in a total volume of 1.5 mL at 50 °C under static conditions (RAC for 4 h, FP for 24 h) supplemented with 2 mg/mL of purified MMPL or MPKL, and 25 μg of enzyme for RAC or 100 μg of enzyme for FP in 100 mM potassium phosphate (pH 7.5). Ampicillin and zeocin (each at 25 μg/mL) were added to the reaction solutions to prevent microbial contamination. Samples (50 μL) were taken out at regular intervals. The reaction was stopped via boiling (10 min). Samples were centrifuged at 10,000*g* for 10 min. The residues and liquids were collected and used to assay reducing sugars in insoluble and soluble fractions, respectively.

## Results

### Expression and purification of EG1 and its variants

**Fig. 1:**
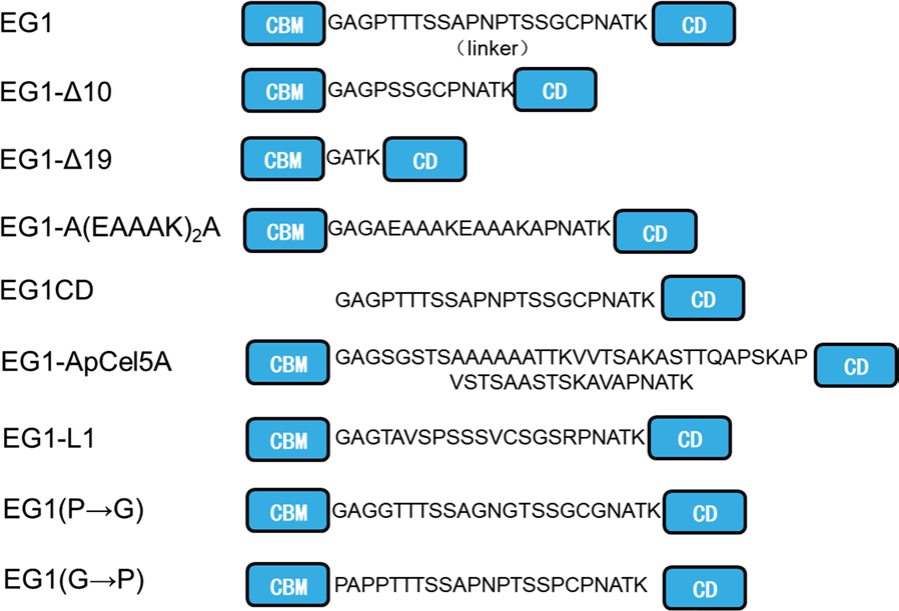
Schematic structures of EG1 and its variants

A graphical representation of EG1 and its variants is shown in Fig. 1. EG1-Δ10 and EG1-Δ19 variants had 10-amino acids and 19-amino acids deletions in the linker region of EG1, respectively. For EG1-A(EAAAK)_2_A, EG1-ApCel5A, and EG1-L1, their linker regions were replaced with a rigid linker A(EAAAK)_2_A, a linker from GH5 endoglucanase (ApCel5A) of *Aureobasidium pullulans* (GenBank AEM23896), and a partial linker from GH5 protein of *Phlebiopsis gigantea* 11061_1 CR5-6 (Sequence ID: KIP10991.1), respectively. For EG1(P→G) and EG1(G→P), the P or G residue in the linker region of EG1 was substituted by a G or P residue, respectively. EG1 and its variants were functionally expressed in *P. pastoris.* The recombinant proteins with a 6× His-tag at the C-terminus were purified by affinity chromatography in a one-step procedure using Ni–NTA agarose gel, without final removal of the His-tag. The molecular weights for purified recombinant proteins were estimated to be approximately 40 kDa (EG1), 39 kDa (EG1-Δ10), 37 kDa (EG1-Δ19), 39 kDa (EG1-A(EAAAK)_2_A), 42 kDa (EG1-ApCel5A), 36 kDa (EG1CD), 40 kDa (EG1-L1), and 40 kDa for EG1(P→G) and EG1(G→P) as shown in Additional file 2: Fig. S1. The wild-type enzyme contains two inherent putative *N*-glycosylation sites (Asn–X–Thr/Ser, in which X is not proline; one site (NAT) at the linker region close to the CD, another (NNT) at the CD) and several possible *O*-glycosylation sites [36]. Both the two *N*-glycosylation sites remained in the variants with the exception of EG1-Δ19 and EG1(P→G). For EG1-Δ19, the *N*-glycosylation site (NAT) at the linker region was removed, while for EG1(P→G), a new *N*-glycosylation site was introduced at the linker region due to the substitution of NPT to NGT (Fig. 1). So, all recombinant proteins had *N*-glycosylation as indicated by their relatively higher molecular weights compared to their predicted values on SDS-PAGE (Additional file 2: Fig. S1).

### Substrate specificity of EG1 and its variants

The substrate specificities of EG1 and its variants on various cellulosic substrates are shown in Table 1. EG1-Δ10 and EG1-Δ19 exhibited 78.7% and 15.9% of EG1 activity on the soluble substrate CMC-Na, 79.1% and 26.2% on RAC, and 69.9% and 53.7% on FP, respectively. EG1CD displayed 91.2%, 83.1%, and 86.4% of EG1 activity on CMC-Na, RAC, and FP, respectively. EG1(P→G) showed 87.0%, 59.7%, and 81.5% of EG1 activity on CMC-Na, RAC, and FP, respectively. EG1(G→P) and EG1-L1 had no significant changes in specific activities on CMC, RAC, and FP compared to EG1. EG1-ApCel5A displayed slightly higher activity than EG1 on CMC-Na (111.2%), but similar activities as EG1 on RAC and FP. Interestingly, EG1-A(EAAAK)_2_A with a synthetic rigid linker had similar activity on CMC-Na compared to EG1, but its activity towards RAC and FP was reduced to 73.5% and 90.8% of EG1, respectively.

### Processivity of EG1 and its variants

**Fig. 2:**
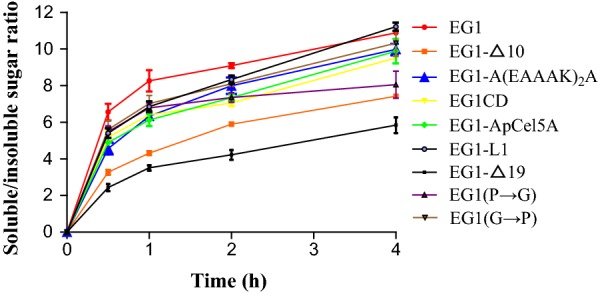
Processivity from PASC by EG1 and its variants

**Fig. 3:**
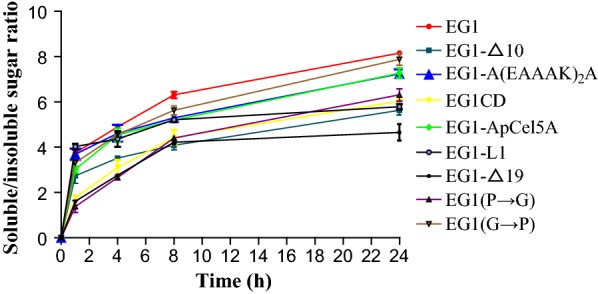
Processivity from FP by EG1 and its variants

EG1-L1 displayed the highest soluble/insoluble product ratios on RAC while EG1 gave the highest soluble/insoluble product ratios on FP (Figs. 2, 3). For EG1-L1, the ratio of soluble to insoluble reducing sugar fractions increased from 5.4 to 11.2 on RAC, and 4.0 to 5.8 on FP, as the incubation time was prolonged from 0.5 h to 4 h, and 1 h to 24 h, respectively. For EG1, the corresponding ratio increased from 6.6 to 10.9 on RAC, and 3.8 to 8.2 on FP, respectively (Figs. 2, 3). EG1-Δ19 displayed the lowest soluble/insoluble product ratios on both RAC and FP; the corresponding ratios increased from 2.4 to 5.8 on RAC and 1.6 to 4.7 on FP, respectively. Other variants also showed relatively lower ratios than EG1 (Additional file 3: Table S2 and Additional file 1: Table S3). The reduced processivity of EG1-Δ19 and other variants resulted in a decrease in reduced soluble sugars released from substrates, with the exception of EG1-ApCel5A, which released more total reducing sugars than EG1 (data not shown). The total reducing sugars generated from RAC by EG1-ApCel5A were 586.6 μg after 4 h of hydrolysis, approximately 9.9% more than EG1. Meanwhile, the total reducing sugars released from FP reached 278.6 μg after 24 h of hydrolysis, approximately 18.6% more than EG1.

### Lignin-binding affinity of EG1 and its variants

**Fig. 4:**
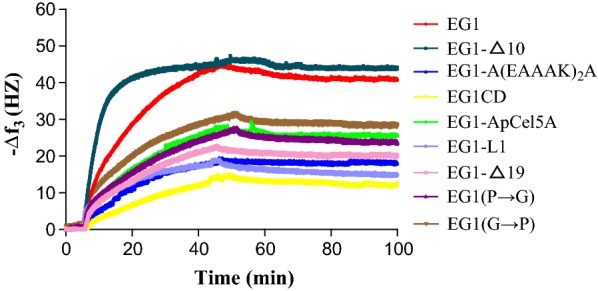
Adsorption values of EG1 and its variants on MMPL lignin film

In this study, the adsorption and desorption behaviors of enzymes on lignin film were analyzed using QCM-D. The instrument simultaneously captures the real-time changes in the resonance frequency (Δ*f*) and energy dissipation (Δ*D*) when the mass of an oscillating piezoelectric crystal changes upon an increase/decrease in the mass of the crystal surface due to added/deduced protein [37]. The changes in resonance frequency (Δ*f*) upon the feeding of enzymes on the lignin-coated sensor surfaces are shown in Fig. 4. The energy dissipation (Δ*D*) refers to the frictional losses that lead to dampening of the oscillation depending on the viscoelastic properties of the material [37]. The change in energy dissipation (Δ*D*) was low (< 3) for the enzymes studied, indicating that the enzyme adsorption resulted in a thin rigid layer (data not shown). Remarkably, significant differences in QCM frequency profiles were observed for EG1-Δ10 and EG1 compared to other variants. EG1-Δ10 displayed a substantially faster initial adsorption rate on the lignin film, the maximum adsorption being reached within 20 min compared to all the other variants, which reached the maximum adsorption after 45 min. EG1 also exhibited a significantly quicker adsorption rate than other variants, particularly in the initial stage. Furthermore, both EG1-Δ10 and EG1 had notably higher increases in the negative value of the frequency shift compared to other variants. The adsorption of an enzyme will cause a change in the frequency shifts (Δ*f*) of the oscillating quartz crystal, the amount of adsorbed enzyme being proportional to the negative shift of QCM frequency. The adsorbed masses of enzymes on the lignin films were calculated by QCM frequency shifts (Δ*f*) (Table 2). The maximum binding of EG1 and its variants on lignin films decreased in the following order: EG1-Δ10>EG1>EG1(G→P)>EG1-ApCel5A>EG1(P→G)>EG1-Δ19>EG1-A(EAAAK)_2_A>EG1-L1>EG1CD, on a weight basis. During the buffer wash stage, 5.4–16.6% of bound enzymes were detached from the lignin film (Fig. 4, Table 2), indicating that most of the adsorption of EG1 and its variants onto lignin film appeared to be irreversible. Among EG1 and its variants, EG1-L1 was absorbed in the smallest quantities onto the lignin film when compared to other EGs with CBM1 and was detached the most during the wash stage (16.6% release) (Table 2). EG1-Δ10 had the highest lignin affinity but the lowest reversibility (5.4% release) (Fig. 4, Table 2). As expected, EG1CD had the slowest adsorption rate and the lowest lignin adsorption affinity because of the lack of a CBM.

**Table 2:** Adsorption and desorption values of EG1 and its variants on MMPL lignin films

Enzymes	Max Δ*m* (ng)	Stabilized Δ*m*′ (ng)	Wash off Δ*m*″ (ng)
EG1	264.57	242.4	22.1
EG1-Δ10	275.66	260.79	14.87
EG1-Δ19	132.16	118.78	13.38
EG1-A(EAAAK)_2_A	119.48	105.11	14.37
EG1CD	87.15	80.35	6.8
EG1-ApCel5A	166.77	153.86	12.91
EG1-L1	106.40	88.75	17.65
EG1-(P→G)	158.16	141.32	16.84
EG1-(G→P)	182.52	169.93	12.59

### Effect of lignin on EG1 and its variant enzyme activity

In general, the activities of all EG1 and its variants against various cellulosic substrates were somewhat inhibited by the presence of two types of lignin (Table 3). MMPL lignin showed the highest inhibitory effect on EG1-Δ10 activity (21.1%) towards CMC-Na, followed by EG1-L1 (15.1%), EG1 (14.8%), EG1(G→P) (14.9%), EG1(P→G) (13.9%), EG1-ApCel5A (12.3%), EG1-A(EAAAK)_2_A (11.6%), EG1-Δ19 (10.3%), and EG1CD (2.9%) (Table 3). For the insoluble substrate RAC, MMPL lignin reduced EG1-Δ10 activity by nearly 24.0%, followed by EG1(G→P) (14.1%), EG1 (13.8%), EG1(P→G) (12.1%), EG1-ApCel5A (11.6%), EG1-L1 (10.3%), EG1-Δ19 (9.9%), EG1CD (8.3%), and EG1-A(EAAAK)_2_A (7.3%) (Table 3). MMPL lignin also displayed a weak inhibitory effect on the activities of EG1 and its variants towards the insoluble substrate FP. The activities of EG1-Δ10, EG1, EG1(G→P), EG1(P→G), EG1-Δ19, EG1-A(EAAAK)_2_A, EG1-ApCel5A, EG1-L1, and EG1CD towards FP were reduced by 18.4%, 10.4%, 9.5%, 7.4%, 7.1%, 5.9%, 4.1%, 3.2%, and 2.9%, respectively (Table 3). A similar inhibition pattern was observed for all variants against various cellulosic substrates when supplemented with MPKL lignin (Table 3).

**Table 3:** Specific activity of EG1 and its variants on different substrates with added lignin

Enzymes	Specific activity (U/μmol)					
	MMPL lignin			MPKL lignin		
	CMC-Na	RAC	FP	CMC-Na	RAC	FP
EG1	1261.30 ± 16.89	495.17 ± 6.19	7.26 ± 0.15	1292.53 ± 27.05	316.61 ± 0.52	6.65 ± 0.65
EG1-Δ10	918.91 ± 18.36	345.24 ± 9.91	4.62 ± 0.17	978.45 ± 39.16	234.99 ± 0.61	4.41 ± 0.33
EG1-Δ19	210.78 ± 10.63	135.68 ± 3.53	4.04 ± 0.15	190.43 ± 23.57	128.18 ± 8.91	3.67 ± 0.51
EG1-A(EAAAK)_2_A	1327.70 ± 27.81	399.80 ± 1.25	7.06 ± 0.05	1267.59 ± 38.58	353.40 ± 9.23	6.77 ± 0.33
EG1CD	1311.03 ± 21.48	437.39 ± 4.17	6.80 ± 0.09	1170.26 ± 42.84	338.60 ± 5.10	6.54 ± 0.46
EG1-ApCel5A	1444.70 ± 44.14	506.02 ± 5.51	7.06 ± 0.05	1463.72 ± 41.71	464.94 ± 4.29	6.44 ± 0.24
EG1-L1	1243.79 ± 32.34	427.88 ± 5.34	7.05 ± 0.06	1267.50 ± 20.34	427.4 ± 10.72	6.51 ± 0.48
EG1-(P→G)	1110.46 ± 19.56	301.56 ± 18.90	6.11 ± 0.52	1044.81 ± 27.98	282.03 ± 1.23	6.11 ± 0.77
EG1-(G→P)	1356.56 ± 31.45	472.45 ± 31.88	8.10 ± 0.77	1273.92 ± 33.19	419.82 ± 2.11	8.02 ± 0.15

Values shown are means of triplicate determinations ± standard error (SE)

### Influence of lignin on enzymatic hydrolysis of RAC and FP by EG1 and its variants

**Fig. 5:**
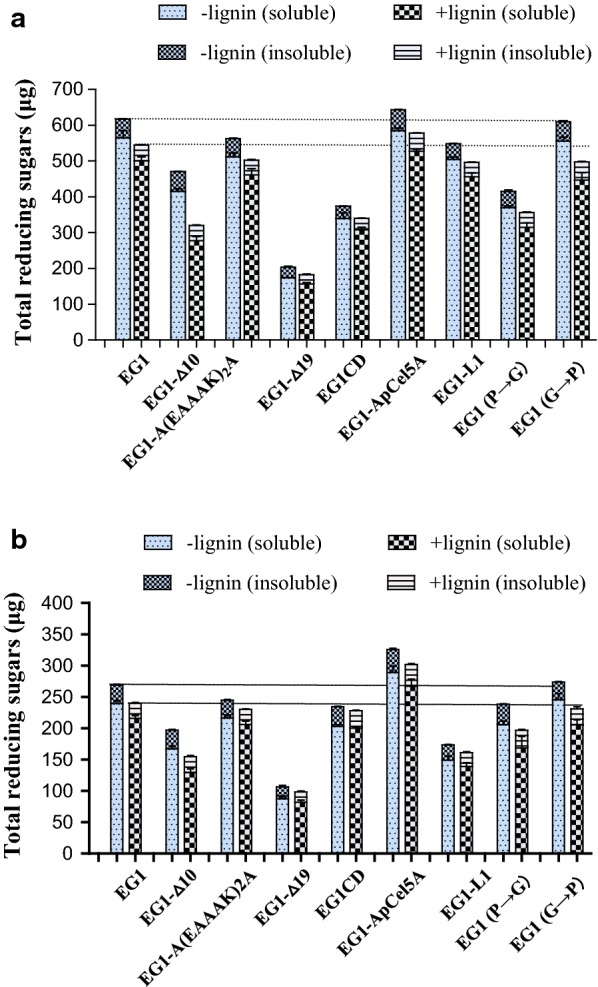
Inhibitory effect of MMPL lignin on enzymatic hydrolysis by EG1 and its variants. **a** Enzymatic hydrolysis of RAC; **b** enzymatic hydrolysis of FP

**Fig. 6:**
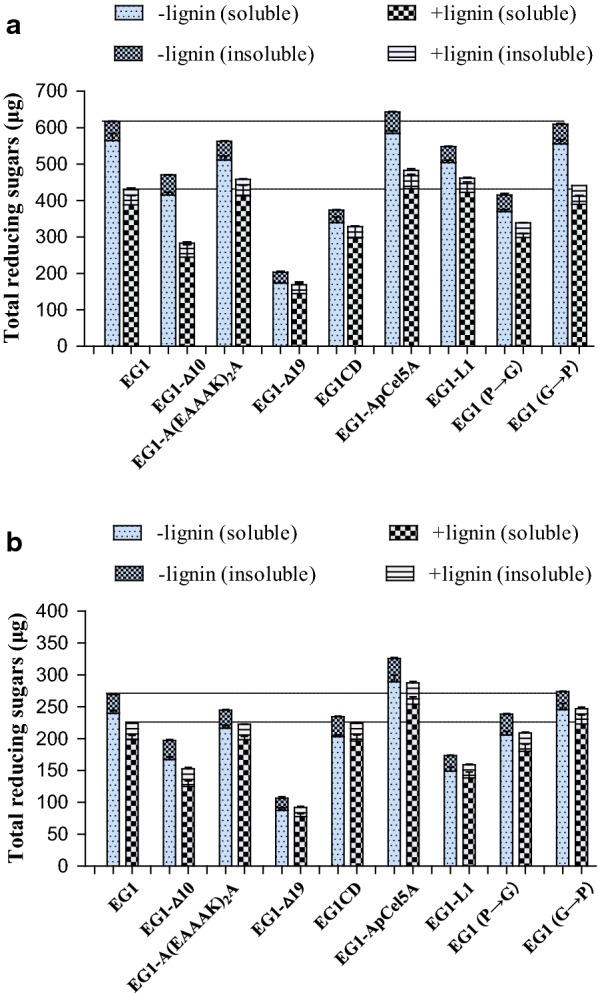
Inhibitory effect of MMKL lignin on enzymatic hydrolysis by EG1 and its variants. **a** Enzymatic hydrolysis of RAC; **b** enzymatic hydrolysis of FP

The inhibitory effects of two types of lignin on the enzymatic hydrolysis of RAC and FP by EG1 and its variants were studied (Figs. 5, 6). The reaction times were 4 h and 24 h for RAC and FP, respectively. As shown in Fig. 5a, EG1-Δ10 and EG1 RAC hydrolysis efficiencies were reduced by 32% and 11.8%, respectively, in the presence of MMPL lignin, with the least pronounced inhibition (4.4%) recorded for EG1CD. Meanwhile, other variants’ hydrolysis efficiencies were only reduced by less than 10%. When the inhibitory effect of MMPL lignin was tested in the FP hydrolysis reaction (Fig. 5b), the highest and the lowest inhibitions of hydrolysis efficiency were also recorded for EG1-Δ10 and EG1CD, respectively. EG1-Δ10 had its hydrolysis efficiency reduced by 21.9%, while EG1CD had its hydrolysis efficiency reduced by 2.6%. EG1-(P→G), EG1-(G→P), EG1, EG1-Δ19, EG1-ApCel5A, EG1-L1, and EG1-A(EAAAK)_2_A had reductions of 17.5%, 15.5%, 13%, 7.4%, 7.4%, 7.0%, and 5.9% in hydrolysis efficiencies, respectively. When MPKL lignin was used to test the inhibitory effect of lignin on the enzymatic hydrolysis of RAC and FP, its inhibitory effect was more prominent than that of MMPL lignin (Fig. 6a, b). The relative inhibition reached 12.1% to 39.8% on RAC, and 4.4% to 22.6% on FP, respectively. But similarly, the highest and the lowest inhibitions of hydrolysis efficiency were also recorded for EG1-Δ10 and EG1CD, respectively. It is worth mentioning that compared to other EGs, EG1-ApCel5A displayed the best hydrolysis capacity on RAC and FP regardless of whether lignin was present. After a 4 h or 24 h hydrolysis in the presence of MMPL, EG1-ApCel5A generated 577.9 μg and 301.9 μg of total reducing sugars from RAC and FP, respectively, which were 6.2% and 30.1% more than from EG1 (Fig. 5). When hydrolyzed in the presence of MPKL, EG1-ApCel5A generated 483 μg and 287.4 μg of total reducing sugars from RAC and FP, respectively, which were 12.5% and 28.2% more than from EG1 (Fig. 6). However, for other variants, the overall enzymatic hydrolysis efficiency was similar or lower than that of EG1 due to the obvious decrease in activity against RAC and FP after mutation.

## Discussion

The glycosyl hydrolase family-5 (GH5) endo-β 1,4-glucanases are ubiquitous enzymes in fungi and are key components of enzyme cocktails for lignocellulosic biomass conversion [38]. The lignin adsorption capacities of EGs are less than those of CBHs, but it has been found that the hydrolysis efficiencies of EGs are far more influenced by lignin binding compared to CBHs [20, 39]. Both the CBM and CD domains of EGs contribute to the enzyme adsorption to lignin, mainly through hydrophobic interactions and electrostatic or hydrogen-bonding interactions [17, 19]. However, previous research has revealed that intact EG enzymes exhibit much higher affinities on lignin than either CBM or CD deletion mutants [12, 19], suggesting that the two domains may participate synergistically in the adsorption to lignin. The linkers of fungal EGs typically have lengths of 20–50 amino acid residues and low sequence similarity [38]. A number of studies have shown that linkers are often not only flexible connectors but also have functional roles in the activity and cellulose-binding of cellulases. Previous reports have demonstrated that the length, amino acid content, and stiffness or flexibility of the linkers play important roles in cellulase catalytic properties [40–44]. The linker length also plays a key role in the processivity of cellobiohydrolase I, because shortening or deleting the linker reduces enzymatic activity on crystalline cellulose [29, 45]. More recently, it has been shown that removing the predicted glycosylation sites in the linker region leads to increased lignin affinity, while adding predicted glycosylation sites decreases lignin affinity [17].

Despite the wide recognition that the linker has functional roles other than domain connectivity, the significance of the linker in endoglucanases is still less understood than the roles of CBM and CD, particularly with respect to their influence on lignin adsorption and processivity. Here, we observed the effects of different linkers on the lignin adsorption and processivity of a GH5 endoglucanase (EG1) from *V. volvacea*. EG1 belongs to the GH5_2 subfamily of endo-β-1,4-glucanases (EC 3.2.1.4) and contains a 36-aa CBM at its N-terminus and a 307-aa CD at its C-terminus connected with a flexible 23-aa linker. N-Linked glycans in linkers have a significant influence on the properties of cellulases [46, 47]. In this study, the inherent putative *N*-glycosylation site in the linker region was retained in all the variants except EG1-Δ19 and EG1(P→G) in order to diminish the impact caused by different *N*-glycosylation levels on the linker or on lignin affinity and processivity. The shorter variant EG1-Δ10 had its processivity dramatically reduced on RAC and FP and had reduced catalytic activity, but higher lignin affinity than the wild-type enzyme. However, further deletion of the linker (EG1-Δ19) resulted in significant reductions of the lignin affinity and processivity as well as catalytic activity. The EG1-ApCel5A linker consists of 52 amino acid residues (twice as much as EG1); it exhibited slightly lower processivity on RAC and FP than EG1, but its lignin affinity was notably reduced to only 63% of that of EG1. These results suggest that proper spatial separation of the two domains is essential for lignin affinity and processivity, yet the optimal length is different. Similar to other cellulases, the linker of EG1 is also rich in proline (4) and glycine (2). The proline residues enhance linker rigidity and enable extended conformations, while glycine promotes additional flexibility to allow for the proper orientation between domains [31, 48]. To study the influence of these compositions in lignin binding and processivity, three variants, EG1-L1, EG1-(P→G), and EG1-(G→P), were constructed that have the same linker lengths as EG1 but differences in amino acid composition. Interestingly, all of them displayed prominent degrees of decline in lignin binding and moderate decreases in processivity. With more detailed analysis, the substitution of G with P had a more significant influence than the substitution of P with G, since EG1-(G→P) was adsorbed to a higher extent and with greater strength on lignin film than EG1-(P→G). To better understand the influence of the linker stiffness on lignin binding and processivity, EG1-A(EAAAK)_2_A with a synthetic rigid linker was constructed [49]. The processivity of EG1-A(EAAAK)_2_A was slightly less than that of EG1, while the lignin binding was significantly reduced to 45.2% of EG1, indicating that proper flexibility is relevant for lignin binding. These results suggested that either the length or the specific amino acid composition (and probably the resulting changes of the charge and hydrophobicity) of the linker may have a prominent influence on the lignin binding of the enzyme tested in this study, since all variants except EG1-Δ10 had a notable decrease in lignin affinity. Comparatively, the processivity may depend primarily on the length of the linker and less so on the specific amino acid composition, as a notable reduction of processivity was observed for EG1-Δ10 and EG1-Δ19, while other amino acid substitution variants had only moderate decreases in processivity. The importance of linker length for cellulase function is well documented [50, 51]. It has been proposed that the linker lengths between structured domains are optimized based on the GH domain and CBM type [31]. The longer linker may promote a flexible extended conformation between domains and may optimize the interaction between structured domains [52]. These observations are quite relevant in processive cellulases, in which the inter-domain interaction may be especially important for their function [53]. Since all enzymes in this study contained an inherent *N*-glycosylation site at the CD, and most of them also retained the *N*-glycosylation site at the linker [except EG1-Δ19 and EG1-(P→G)], it is reasonable to believe that these enzymes have similar levels of *N*-glycosylation. Thus, we may conclude that the modulation of lignin binding and processivity among EG1 and its variants is not related to *N*-glycosylation of the linker. However, it is worth noting that the impact of *O*-glycosylation should not be excluded and need to be investigated [17, 31].

Since a notable decrease in lignin binding was observed in the variants, we further investigated how much this effect may contribute to relieving the lignin inhibition and improving their catalytic activity and hydrolysis efficiency. Consistent with our expectations, the lignin affinity and lignin inhibition of the variants were highly correlated in general, although several variants displayed some differences in reductions of lignin binding or lignin inhibition. EG1-Δ10 and EG1, harboring the first and second highest lignin-binding affinities, were also accompanied by the first and second highest inhibitory effects of lignin on activity and hydrolysis efficiency, respectively. The finding that EG1CD had one-third of the wild-type lignin-binding affinity of EG1 and was also inhibited by supplemental lignin is consistent with previous reports showing that lignin binding is mainly driven by the CBM, but the CD is also responsible [10, 12, 15–19]. However, removing the CBM from cellulases is not a feasible strategy to reduce the inhibitory effect of lignin because of the dramatic loss of catalytic ability without a CBM [19]. Consequently, EG1-ApCel5A, which retained higher specific activity but much weaker lignin-binding affinity, outperformed the wild-type enzyme in the enzymatic hydrolysis of RAC and FP with added lignin. From 28.2 to 30.1% more total reducing sugars were generated from FP by EG1-ApCel5A in the presence of lignin compared to EG1. Non-productive adsorption of cellulases to lignin causes the overall cost to increase during lignocellulosic biomass hydrolysis. It would be helpful to reduce the inhibitory effect of lignin on enzymatic hydrolysis by engineering a weak lignin-binding cellulase [17, 54, 55]. Compared to most cellulase engineering projects on CBMs, our work suggests that engineering cellulase in the linker region may retain the catalytic properties of the enzyme but significantly reduce the lignin-binding affinity, improving the overall performance of enzymatic hydrolysis in the presence of lignin. It is also worth noting that not all the linker modifications improved the overall enzymatic hydrolysis performance with added lignin, even though lignin adsorptions were notably reduced due to the simultaneous decrease of catalytic activity. Since the linkers do not serve simply as tethers between structured domains, but rather as important contributors to enzyme activity in multiple ways [47], a more detailed understanding of the role of the linker region in lignin adsorption and catalysis in processive endoglucanases is still needed.

## Conclusions

In conclusion, our current results highlight the relevance of the linker region in the lignin adsorption and processivity of processive endoglucanases. The influence of the linker on processivity may depend primarily on the length of the linker and less so on the specific amino acid composition, yet both of these contribute to lignin binding. The lignin inhibition of the enzymatic hydrolysis of the variants was, in general, proportional to the lignin affinity, suggesting a high correlation. A variant EG1-ApCel5A, obtained by inserting a long, flexible linker, outperformed the wild-type enzyme in the enzymatic hydrolysis of RAC and FP with added lignin due to significantly reduced lignin binding with the retention of catalytic properties. Since EG1 is a typical GH5 processive endoglucanase with a very common modular architecture, our findings promote a better understanding of the influence of the linker region on lignin adsorption and the catalytic capacities of GH5 processive endoglucanases, and may be useful for the development of newly engineered weak lignin-binding cellulases.

## Additional files

**Additional file 1: Table S1.** Primes used in this study.

**Additional file 2: Figure S1.** SDS-PAGE of EG1 and its variants. Lane M: protein markers; lanes 1–9: EG1-Δ10, EG1-Δ19, EG1-A(EAAAK)_2_A, EG1CD, EG1-ApCel5A, EG1-L1, EG1, EG1-(P→G) and EG1-(G→P), respectively.

**Additional file 3: Table S2.** Processivity on PASC by EG1 and its variants.

**Additional file 4: Table S3.** Processivity on FP by EG1 and its variants.

## Abbreviations

CBH: cellobiohydrolase
EG: endoglucanase
LPMO: lytic polysaccharide monoxygenase
GH: glycosyl hydrolase
CBM: carbohydrate-binding module
CD: catalytic domain
Mw: weight average molecular weights
CMC-Na: sodium carboxymethylcellulose
RAC: regenerated amorphous cellulose
FP: filter paper
SDS-PAGE: sodium dodecyl sulfate polyacrylamide gel electrophoresis
MMPL: milled Masson pine lignin
MPKL: Masson pine kraft lignin
QCM-D: quartz crystal microgravimetry with dissipation monitoring
