# Supplementary material for: Altering the linker in processive GH5 endoglucanase 1 modulates lignin binding and catalytic properties
Source: Biotechnol Biofuels. 2018 Dec 18;11:332. doi: 10.1186/s13068-018-1333-3 (PMC6297974; doi:10.1186/s13068-018-1333-3)
Supplement: Supplementary file 1 — Additional file 1: Table S1. Primes used in this study. [file 13068_2018_1333_MOESM1_ESM.docx]

**Additional file 1**

Table S1 Primes used in this study

| Enzymes | Fragments | Primer pairs（5’-3’） | |
| --- | --- | --- | --- |
| EG1-△10 | CBM | 5’- | AAAACTGCAGCCGTCCCAGTATGGGGACAAT |
|  |  | 3’- | GGCAGCCACTGGAAGGTCCGGCGCCAGG |
|  | CD | 5’- | cctggcgccggaccttccagtggctgcc |
|  |  | 3’- | GCTCTAGAGCCACGAATGGTTTCAAAGC |
| EG1-△19 | CBM | 5’- | AAAACTGCAGCCGTCCCAGTATGGGGACAAT |
|  |  | 3’- | gaatctgaacttggtggcgccaggctggcattggtG |
|  | CD | 5’- | Caccaatgccagcctggcgccaccaagttcagattc |
|  |  | 3’- | GCTCTAGAGCCACGAATGGTTTCAAAGC |
| EG1-A(EAAAK)_2_A | CBM | 5’- | AAAACTGCAGCCGTCCCAGTATGGGGACAAT |
|  |  | 3’- | AGCTTCCTTAGCAGCAGCTTCCTTAGCAGCAGCTTCAGCtccggcgcc |
|  | CD | 5’- | Ccgaatgccaccaagttcagattcttcggtgtcaac |
|  |  | 3’- | GCTCTAGAGCCACGAATGGTTTCAAAGC |
| EG1-ApCel5A | CBM | 5’- | AAAACTGCAGCCGTCCCAGTATGGGGACAAT |
|  |  | 3’- | ctgagtagtgctggccttggccgaggtcacaaccttggtggtggcagcagcagcagcagcagaggtggaaccggaaccagctccaggctggcattg |
|  | CD | 5’- | agcactactcaggctccttccaaggctcctgtctctactagcgccgcctctactagcaaggctgttgccccgaatgccacc |
|  |  | 3’- | GCTCTAGAGCCACGAATGGTTTCAAAGC |
| EG1CD | CD | 5’- | AAAACTGCAGGCGCCGGACCTACGACAA |
|  |  | 3’- | GCTCTAGAGCCACGAATGGTTTCAAAGC |
| EG1-L1 | CBM | 5’- | AAAACTGCAGCCGTCCCAGTATGGGGACAAT |
|  |  | 3’- | AGTAGTAGTTGGAGTAGAAGTAGTGGGTGCGCTGCT |
|  | CD | 5’- | ACTACTTCTACTCCAACTACTACTGCTACTTCTCCATCTAaccccacc |
|  |  | 3’- | GCTCTAGAGCCACGAATGGTTTCAAAGC |
| EG1(P→G) | Full plasmid | 5’- | ggaGGCacgacaaccagcagcgcaGGCaacGGCacctccagtggctgcGGCaat |
|  |  | 3’- | attGCCgcagccactggaggtGCCgttGCCtgcgctgctggttgtcgtGCCtcc |
| EG1(G→P) | Full plasmid | 5’- | cctCCGgccCCGcctacgacaaccagcagcgcacccaaccccacctccagtccgtgc |
|  |  | 3’- | gcacggactggaggtggggttgggtgcgctgctggttgtcgtaggCGGggcCGGagg |

Notes: CBM fragment contains a CBM and a partial linker region; CD fragment contains CD and a partial linker region.
