# Supplementary material for: Altering the linker in processive GH5 endoglucanase 1 modulates lignin binding and catalytic properties
Source: Biotechnol Biofuels. 2018 Dec 18;11:332. doi: 10.1186/s13068-018-1333-3 (PMC6297974; doi:10.1186/s13068-018-1333-3)
Supplement: Supplementary file 2 — Additional file 2: Figure S1. SDS-PAGE of EG1 and its variants. Lane M: protein markers; lanes 1–9: EG1-Δ10, EG1-Δ19, EG1-A(EAAAK)2A, EG1CD, EG1-ApCel5A, EG1-L1, EG1, EG1-(P→G) and EG1-(G→P), respectively. [file 13068_2018_1333_MOESM2_ESM.docx]

**Additional file 2**

M 1 2 3 4 5 6 M 7 8 9

100

30

25


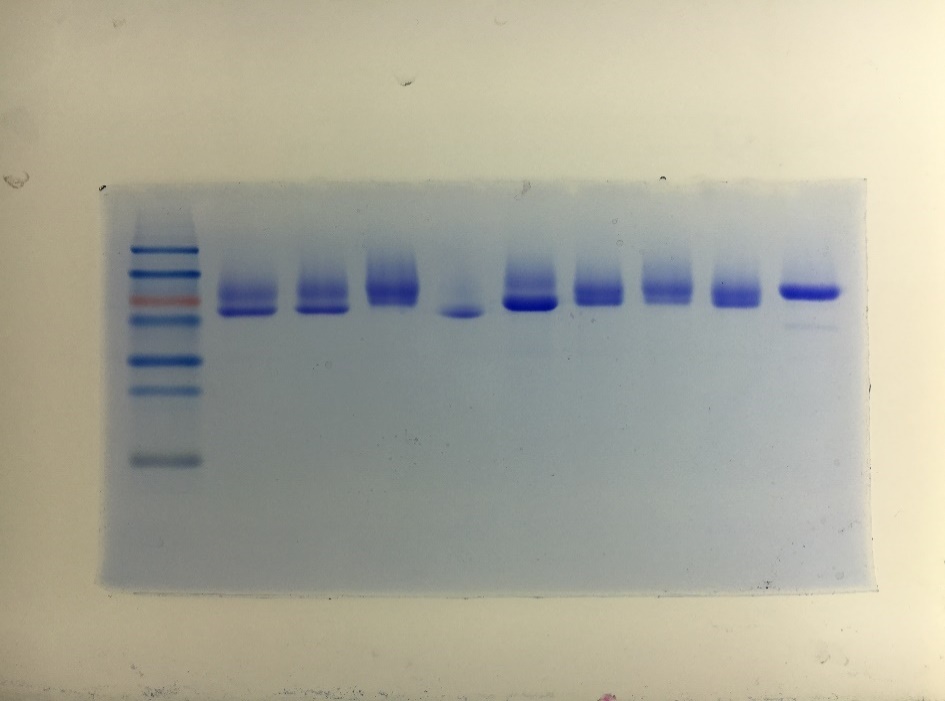


kDa

70

50

40

14


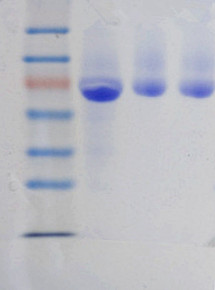


**Fig. S1** SDS-PAGE of EG1 and its variants. Lane M: protein markers; lanes 1–9: EG1-Δ10, EG1-Δ19, EG1-A(EAAAK)_2_A, EG1CD, EG1-ApCel5A, EG1-L1, EG1, EG1-(P→G) and EG1-(G→P), respectively.
