# Supplementary material for: Altering the linker in processive GH5 endoglucanase 1 modulates lignin binding and catalytic properties
Source: Biotechnol Biofuels. 2018 Dec 18;11:332. doi: 10.1186/s13068-018-1333-3 (PMC6297974; doi:10.1186/s13068-018-1333-3)
Supplement: Supplementary file 3 — Additional file 3: Table S2. Processivity on PASC by EG1 and its variants. [file 13068_2018_1333_MOESM3_ESM.docx]

Table S2 Processivity on PASC by EG1 and its variants

| Enzymes | Soluble/insoluble sugar ratios | | | |
| --- | --- | --- | --- | --- |
|  | 0.5h | 1h | 2h | 4h |
| EG1 | 6.56±0.45 | 8.26±0.59 | 9.09±0.13 | 10.89±0.06 |
| EG1-△10 | 3.27±0.16 | 4.31±0.13 | 5.90±0.11 | 7.43±0.01 |
| EG1-△19 | 2.44±0.19 | 3.51±0.14 | 4.22±0.27 | 5.84±0.43 |
| EG1-A(EAAAK)_2_A | 4.57±0.32 | 6.33±0.22 | 8.00±0.44 | 9.98±0.32 |
| EG1CD | 5.19±0.31 | 6.41±0.29 | 7.06±0.16 | 9.52±0.11 |
| EG1-ApCel5A | 4.90±0.09 | 6.13±0.33 | 7.35±0.20 | 9.88±0.67 |
| EG1-L1 | 5.41±0.26 | 6.85±0.29 | 8.33±0.22 | 11.22±0.22 |
| EG1-(P→G) | 5.48±0.33 | 6.78±0.25 | 7.36±0.26 | 8.06±0.73 |
| EG1-(G→P) | 5.6± 0.47 | 7.03± 0.43 | 8.01± 0.13 | 10.33± 0.72 |

Values shown are means of triplicate determinations ± standard error (SE).
