# Supplementary material for: Altering the linker in processive GH5 endoglucanase 1 modulates lignin binding and catalytic properties
Source: Biotechnol Biofuels. 2018 Dec 18;11:332. doi: 10.1186/s13068-018-1333-3 (PMC6297974; doi:10.1186/s13068-018-1333-3)
Supplement: Supplementary file 4 — Additional file 4: Table S3. Processivity on FP by EG1 and its variants. [file 13068_2018_1333_MOESM4_ESM.docx]

Table S3 Processivity on FP by EG1 and its variants

| Protein | Soluble/insoluble sugar ratios | | | |
| --- | --- | --- | --- | --- |
|  | 1h | 4h | 8h | 24h |
| EG1 | 3.75±0.16 | 4.90±0.01 | 6.31±0.15 | 8.16±0.00 |
| EG1-△10 | 2.74±0.34 | 3.52±0.03 | 4.09±0.20 | 5.63±0.21 |
| EG1-△19 | 1.62±0.04 | 2.76±0.01 | 4.22±0.10 | 4.66±0.36 |
| EG1-A(EAAAK)_2_A | 3.69±0.25 | 4.62±0.37 | 5.30±0.18 | 7.24±0.20 |
| EG1CD | 1.78±0.04 | 3.10±0.34 | 4.43±0.33 | 6.03±0.14 |
| EG1-ApCel5A | 3.02±0.13 | 4.53±0.31 | 5.19±0.07 | 7.27±0.22 |
| EG1-L1 | 4.03±0.13 | 4.37±0.34 | 5.21±0.10 | 5.79±0.12 |
| EG1-(P→G) | 1.38± 0.27 | 2.66± 0.10 | 4.54± 0.04 | 7.10± 0.27 |
| EG1-(G→P) | 3.32± 0.08 | 4.56± 0.09 | 5.63± 0.20 | 7.88± 0.26 |

Values shown are means of triplicate determinations ± standard error (SE).
